# Pangenome-Guided Reverse Vaccinology and Immunoinformatics Approach for Rational Design of a Multi-Epitope Subunit Vaccine Candidate Against the Multidrug-Resistant Pathogen *Chromobacterium violaceum*: A Computational Immunopharmacology Perspective

**DOI:** 10.3390/ph19010029

**Published:** 2025-12-22

**Authors:** Khaled S. Allemailem

**Affiliations:** Department of Medical Laboratories, College of Applied Medical Sciences, Qassim University, Buraydah 51452, Saudi Arabia; k.allemailem@qu.edu.sa; Tel.: +966-16-301-0555

**Keywords:** *Chromobacterium violaceum*, multi-epitope vaccine, pangenome, multidrug resistance, reverse vaccinology, subtractive proteomics, immunoinformatics, vaccine design, immunopharmacology

## Abstract

**Background**: *Chromobacterium violaceum* is an emerging multidrug-resistant (MDR) Gram-negative bacterium associated with severe septicemia, abscess formation, and high mortality, particularly in immunocompromised individuals. Increasing antimicrobial resistance and the absence of approved vaccines underscore the urgent need for alternative preventive strategies. Traditional vaccine approaches are often inadequate against genetically diverse MDR pathogens, prompting the use of computational immunology and reverse vaccinology for vaccine design. **Objectives**: This study aimed to design and characterize a novel multi-epitope subunit vaccine (MEV) candidate against *C. violaceum* using a comprehensive pangenome-guided subtractive proteomics and immunoinformatics pipeline to identify conserved antigenic targets capable of eliciting strong immune responses. **Methods**: Comparative genomic analysis across eight *C. violaceum* strains identified 3144 core genes. Subtractive proteomics filtering yielded two essential, non-homologous, surface-accessible, and antigenic proteins—penicillin-binding protein 1A (Pbp1A) and organic solvent tolerance protein (LptD)—as vaccine targets. Cytotoxic T-lymphocyte (CTL), helper T-lymphocyte (HTL), and B-cell epitopes were predicted and integrated into a 272-amino-acid MEV construct adjuvanted with human β-defensin-4A using optimal linkers. The construct was evaluated through structural modeling, molecular docking with TLR4, molecular dynamics simulation, immune simulation, and in silico cloning into the pET-28a(+) vector. **Results**: The MEV construct exhibited strong antigenicity, non-allergenicity, and non-toxicity, with stable tertiary structure and favorable physicochemical properties. Docking and dynamics simulations demonstrated high binding affinity and stability with TLR4 (ΔG = −16.2 kcal/mol), while immune simulations predicted durable humoral and cellular immune responses with broad population coverage (≈89%). Codon optimization confirmed high expression potential in *E. coli* K12. **Conclusions**: The pangenome-guided immunoinformatics approach enabled the identification of conserved antigenic proteins and rational design of a promising multi-epitope vaccine candidate against MDR *C. violaceum*. The construct exhibits favorable immunogenic and structural features, supporting its potential for experimental validation and future development as a preventive immunotherapeutic against emerging MDR pathogens.

## 1. Introduction

*Chromobacterium violaceum*, a facultative anaerobic, Gram-negative motile bacillus, has emerged as a rare but highly lethal opportunistic pathogen [1,2]. Widely distributed in soil and stagnant water of tropical and subtropical regions, it is traditionally regarded as an environmental saprophyte [3,4]. However, in susceptible hosts, particularly immunocompromised individuals or those with chronic granulomatous disease, *C. violaceum* infections can rapidly escalate from localized cutaneous lesions or lymphadenitis to fulminant septicemia, hepatic and pulmonary abscesses, and fatal multiorgan failure [4,5,6]. Although reported human cases remain relatively rare, the associated mortality rate is extremely high, underscoring the neglected but significant threat posed by this pathogen [7,8].

The ability of *C. violaceum* to cause severe, rapidly progressive infections is driven by a complex arsenal of virulence factors [9]. Violacein, its characteristic bisindole pigment, while antimicrobial in environmental niches, functions within the host as a cytotoxic metabolite enhancing bacterial survival against oxidative stress and contributing to tissue necrosis [10,11]. The secretion of violacein and other virulence factors is facilitated by outer membrane vesicles (OMVs), tightly regulated by the quorum sensing system CviI/CviR [9,12]. These vesicles enable quorum-controlled biofilm formation, persistence within tissues, and evasion of host immune responses. Biofilm-associated growth further exacerbates persistence and confers significant tolerance to antibiotic treatment [9,13].

Virulence in *C. violaceum* is largely mediated by factors such as type III secretion system (T3SS) effectors, quorum-sensing components, hemolysins, and metalloproteases, which facilitate host invasion and immune evasion. Several of these virulence-associated proteins are highly conserved across clinical and environmental isolates, making them promising antigenic targets for subunit vaccine development [7,14]. Therefore, the present study incorporated virulence-associated core proteins identified through the pangenome analysis, ensuring that potential antigens are functionally linked to pathogenicity.

In parallel, the bacterium encodes a dual Type III Secretion System (T3SS) consisting of the Cpi-1 and Cpi-2 clusters [9,15]. These needle-like structures inject effector proteins into host cells, manipulating inflammasome activation, inducing pyroptotic cell death, and impairing immune clearance [16]. Such immune subversion facilitates bacterial dissemination, systemic invasion, and the high fatality of septicemia [4,9,16].

Treatment of *C. violaceum* infections is further compromised by its antimicrobial resistance profile [6]. Clinical isolates frequently exhibit resistance to β-lactams, macrolides, and aminoglycosides, leaving fluoroquinolones as the most consistently effective therapeutic option [6]. However, emerging resistance trends and limited availability of second-line drugs in resource-constrained regions raise major concerns for future management [17]. Collectively, quorum-regulated virulence, biofilm-mediated persistence, immune evasion, and multidrug resistance highlight the urgent unmet need for effective prophylactic interventions [18,19].

Despite the rising recognition of *C. violaceum* as a human pathogen, there are currently no approved vaccines or targeted immunotherapies. Conventional vaccine platforms, such as whole-cell inactivated or live-attenuated approaches, pose challenges including reversion risks, incomplete protection, and adverse immune reactions [20,21,22]. Subunit vaccines targeting single antigens, while safer, often fail to elicit broad or durable immunity against genetically variable pathogens [23,24]. In contrast, epitope-based multi-epitope vaccines (MEVs) provide a next-generation strategy by integrating multiple conserved and immunodominant epitopes into a modular construct capable of inducing both humoral and cellular immune responses, while minimizing allergenicity and cross-reactivity [25,26,27,28].

Pangenome analysis, which incorporates the total genetic collection of all strains within a species, has become a commanding framework for detecting strain-specific and conserved genes [29,30]. By differentiating core genes from unique ones or dispensable, pangenomic studies deal critical insights into bacterial pathogenicity, evolution, and antigenic diversity [29]. In vaccine development, aiming the conserved core genome guarantees the selection of largely protective antigenic candidates and decreases the likelihood of immune escape [31]. For pathogens, for example, *C. violaceum*, where strain diversity and clinical relevance are developing issues, a pangenome-guided method improves candidate prioritization by focusing on universally conserved, essential proteins most likely to make cross-protective protection.

Recent improvements in computational vaccinology, mainly immunoinformatics based subtractive proteomics and reverse vaccinology have changed vaccine design pipelines [26,28,31,32,33]. Subtractive proteomics allows systematic screening of complete proteomes to identify essential, non-host-homologous, surface-exposed proteins with minimal autoimmune risk [34,35]. Reverse vaccinology leverages genomic data to forecast antigenic candidates without the need for pathogen culturing [22,32]. Immunoinformatics tools allow high-throughput epitope prediction, toxicity, antigenicity evaluation, population coverage analysis, and allergenicity profiling accelerating the identification of capable vaccine candidates [36,37]. These methods have been successfully applied against different pathogens, emphasizing their applicability to neglected and emerging threats such as *C. violaceum*.

In this study, I integrated pangenome analysis, subtractive proteomics, and immunoinformatics to design a novel multi-epitope subunit vaccine against *C. violaceum*. Conserved antigenic proteins were systematically identified, followed by prediction and selection of T-cell and B-cell epitopes. These were assembled into a chimeric construct adjuvanted with human β-defensin-4A and linked for optimal processing and presentation. Structural validation, molecular docking with TLR4, and molecular dynamics simulations confirmed its stability and strong receptor interactions, while immune simulations predicted durable humoral and cellular responses. Codon optimization and in silico cloning further indicated high expression potential in *E. coli* K12. Unlike previous reverse vaccinology studies that relied solely on reference genomes, our approach integrates a pangenome-guided subtractive proteomics pipeline, ensuring the inclusion of conserved antigenic determinants across all known *C. violaceum* strains. This strategy enhances cross-strain protection and minimizes the risk of immune escape. Additionally, our inclusion of human β-defensin-4A as a natural TLR4-activating adjuvant represents a novel adjuvant selection approach in bacterial MEV design. The novelty of this work lies in combining pangenome-guided antigen discovery with immunoinformatics-driven vaccine design against *C. violaceum*, a neglected but clinically significant pathogen. Future directions include recombinant expression, in vitro immunogenicity assays, and in vivo validation to confirm efficacy, safety, and long-term protection. Additionally, integrating MEV strategies with quorum-sensing inhibitors or immune modulators may offer a dual therapeutic avenue to both attenuate bacterial virulence and strengthen host defense mechanisms.

## 2. Results

### 2.1. Pangenome Analysis of C. violaceum

**Figure 1:**
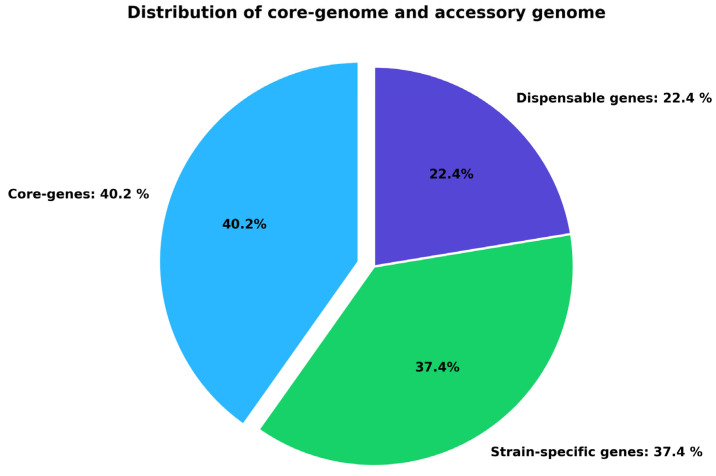
Partitioning of the *C. violaceum* pangenome into core, dispensable, and strain-specific genes. Core genes (40.2%) constitute the conserved genomic component, whereas dispensable (22.4%) and strain-specific (37.4%) genes generate genomic diversity.

The PanExplorer platform and the Roary pipeline conducted a comparative genomic analysis at 80% BLASTP identity on the eight *C. violaceum* strains (Table 1). The assembled pangenome indicated a total gene repertoire that was further subdivided into three broad groups, i.e., core, dispensable and strain-specific genes (Figure 1). Out of all the gene clusters that were found, 3144 (40.2) were found to be core genes, and they were found in all the strains as studied. These central genes are the backbone of the *C. violaceum* genome and they would presumably encode the fundamental functions that are necessary to ensure the survival and virulence of the bacteria. Moreover, 22.4% of the genes were determined as non-essential meaning that they are found in two or more but not in all strains. Most likely, this ratio is the cause of phenotypic variation and adaptation in the niche within the species. Lastly, 37.4 were strain-specific genes which are specific genetic features that may offer strain specific ecological benefit or virulence factors. The similarity between the preserved core genome and accessory variable genome is an indicator of genomic plasticity in *C. violaceum*. Importantly, the identified core proteome was further selected as a subject of downstream subtractive proteomics to be analyzed due to its conservation across all the strains and its application as a source of general drug or vaccine targets.

### 2.2. Subtractive Proteomics

To select potential vaccine candidates from the conserved proteome of *C. violaceum*, a subtractive proteomics pipeline was used to the 3144 core proteins derived from pangenome analysis. The protein sequences were first translated and then filtered for essentiality using the Geptop 2.0 server, which marked 360 proteins as bacterial survival essential. These critical proteins were then subjected to BLASTp analysis against the human proteome (taxid: 9606) to remove homologous sequences and minimize the chances of cross-reactivity. After this filtering procedure, 127 non-homologous critical proteins were left for further analysis. The remaining 233 proteins displayed significant homology with human proteins (≥30% sequence identity and E-value ≤ 1 × 10^−4^) and were thus excluded to minimize potential autoimmune cross-reactivity. This cut-off was consistent with previous subtractive proteomics studies [38,39]. Subcellular localization prediction was carried out with the PSORTb server, and among the shortlisted proteins, 120 were predicted to be cytoplasmic, 25 membrane-localized, and 2 extracellular. Since extracellular and membrane proteins were more accessible to the host immune system, these were given higher priority for immunological analysis. Antigenicity of the selected proteins was assessed by VaxiJen v2.0 with a cut-off of 0.5. Both extracellular proteins possessed antigenic potential and were forecasted to be devoid of transmembrane helices by TMHMM v2.0. Lack of numerous transmembrane domains renders these proteins amenable to subsequent vaccine design since they would be more stable and experimentally accessible (Table 2). These results demonstrate the successful identification of two conserved, extracellular, non-homologous, antigenic proteins without transmembrane domains, being good candidates for further epitope prediction and multi-epitope vaccine (MEV) design against *C. violaceum*. All selected CTL, HTL, and B-cell epitopes demonstrated no significant similarity to human proteins based on BLASTp short-sequence analysis (identity < 30%, E-values above threshold). As the MEV construct incorporates only these filtered epitopes, the complete construct also exhibited no meaningful homology to any human protein sequence, confirming minimal autoimmune risk.

### 2.3. Epitope Prediction

Epitope mapping was conducted on the completed extracellular vaccine candidate proteins to detect cytotoxic T lymphocyte (CTL), helper T lymphocyte (HTL), and linear B-cell (LBL) epitopes relevant for vaccine design. Out of the CTL prediction analysis, 54 epitopes were detected, of which the top 6 epitopes were selected based on their antigenicity, immunogenicity, non-toxicity, and non-allergenicity. These epitopes had the maximum antigenic scores and were selected for incorporation into the vaccine construct (Table 3). For prediction of HTL, 61 epitopes were constructed, and upon screening for antigenicity, non-toxicity, and non-allergenicity, the top 4 epitopes were chosen as the best candidates (Table 4). These epitopes were predicted to bind across multiple HLA-DR alleles and therefore provide broad immunological coverage. In the same vein, linear B-cell epitope prediction generated 40 epitopes, which are surface-accessible regions with the ability to trigger antibody-driven responses. Of these, only the top 2 antigenic, safe, and non-toxic epitopes were selected for use in constructing the final multi-epitope vaccine (Table 5). These CTL, HTL, and LBL epitopes combined become the basis of the constructed MEV construct, bringing balance and vigorous activation of humoral and cell-mediated immune responses (Supplementary Table S1).

### 2.4. Population Coverage Analysis

**Figure 2:**
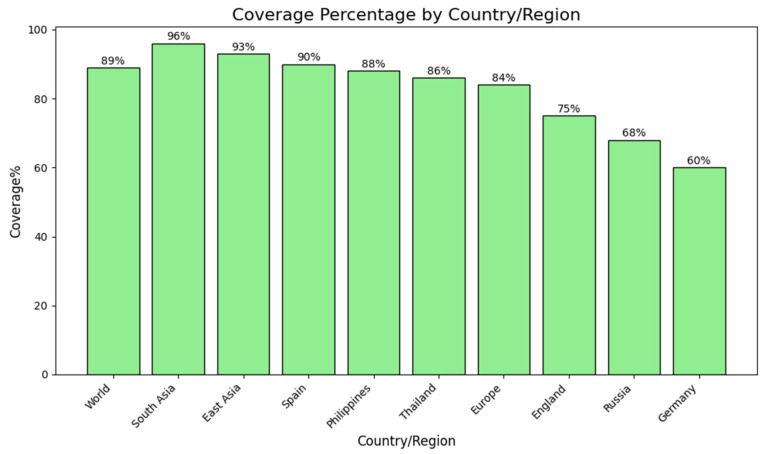
Estimated population coverage of the developed multi-epitope vaccine in global regions. Bar graph showing estimated population coverage percentages of the chosen T-cell epitopes globally and in certain regions and countries. The highest coverage was found in South Asia (96%), followed by East Asia (93%) and Spain (90%). Moderate coverage was found in the Philippines (88%), Thailand (86%), and Europe as a whole (84%). England (75%), Russia (68%), and Germany (60%) had relatively lower coverage rates. These observations suggest that the suggested vaccine construct has wide immunological relevance among various populations, as well as evidence of geographic variation in epitope recognition patterns.

As the distribution of major histocompatibility complex (MHC) alleles differs between geographical locations and ethnic groups, assessment of population coverage is an imperative step in vaccine development. Potential global and regional coverage was estimated for the shortlisted T-cell epitopes. Combined epitopes have an estimated 89% global coverage, indicating their ability to induce immune responses among the vast majority of the world’s population. Regional analysis showed extremely high coverage in South Asia (96%), East Asia (93%), and Southeast Asia (93.06%), followed by Japan (96.19%) and South Korea (90.31%). Conversely, South America (90.02%), Spain (90%), and the Philippines (88%) had moderate coverage levels. Low population coverage was seen in some European populations, including England (75–77%), Russia (68%), and particularly Germany (60%) (Figure 2). These findings suggest that the chosen epitopes give broad coverage for multiple continents, with especially high representation in Asian and South American populations. While coverage within individual European populations was relatively low, broad overall coverage supports the choice of the predicted epitopes as suitable for application in a universal multi-epitope vaccine.

### 2.5. Multi-Epitope Vaccine Construction: Integration of Adjuvant and Linkers for Enhanced Immunogenicity

**Figure 3:**
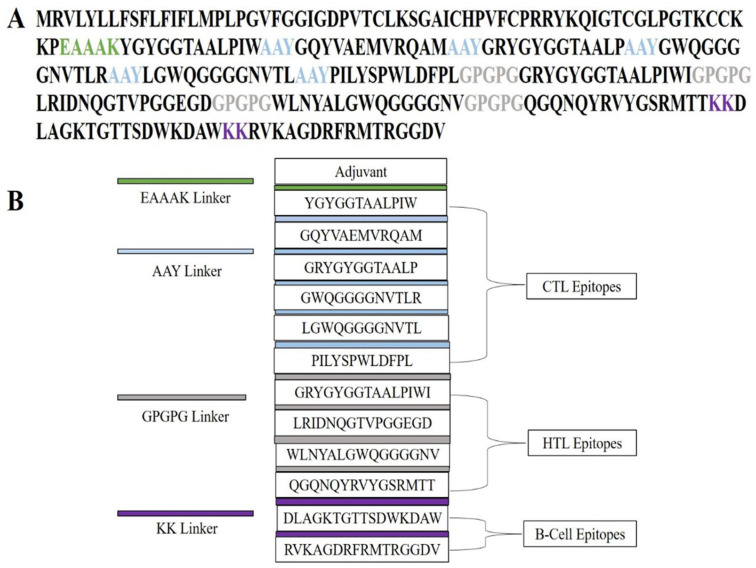
Structural organization of the designed multi-epitope vaccine (MEV) construct. (**A**) Linear depiction of the amino acid sequence of the vaccine construct, wherein functional elements are depicted in varying colors: adjuvant, cytotoxic T-lymphocyte (CTL) epitopes, helper T-lymphocyte (HTL) epitopes, and B-cell epitopes. Particular linkers (EAAAK, AAY, GPGPG, and KK) are labeled in specific colors to show their importance in ensuring stability, flexibility, and accurate epitope presentation. (**B**) Schematic illustration of the modular assembly of the MEV construct. The structure shows the adjuvant placed at the N-terminal, with CTL, HTL, and B-cell epitopes linked by suitable linkers. This organized assembly allows for efficient processing, structural stability, and maximum stimulation of humoral and cellular immune responses.

To elicit a strong and broad immune response, a multi-epitope vaccine (MEV) was constructed by systematically assembling cytotoxic T lymphocyte (CTL), helper T lymphocyte (HTL), and linear B-cell (LBL) epitopes in combination with an immunostimulatory adjuvant. Adjuvants are critical for amplifying antigen recognition and activating the innate arm of the immune system, thereby strengthening overall immunogenicity. In this study, human β-defensin-4A (HBD-4A; NCBI Accession: NP_004933.1) was incorporated as an adjuvant owing to its role as a TLR4 agonist and its ability to potentiate both innate and adaptive immune responses. To ensure appropriate spatial organization and functional preservation of epitopes, specific peptide linkers were incorporated into the construct (see Section 4.5 for methodological details and relevant references). The adjuvant was linked to CTL epitopes via an EAAAK linker, resulting in a structurally separated N-terminal adjuvant domain. CTL epitopes were interconnected using AAY linkers, which are known to favor efficient proteasomal processing and MHC class I presentation, while HTL epitopes were joined using GPGPG linkers to support MHC class II–mediated antigen presentation. Linear B-cell epitopes were separated using bi-lysine (KK) linkers to maintain epitope independence and minimize junctional epitope formation, thereby facilitating effective B-cell receptor recognition. The final MEV construct comprised 272 amino acids, integrating the selected epitopes, linkers, and adjuvant in a modular arrangement designed to reduce steric hindrance and promote proper folding and epitope accessibility. Notably, the complete MEV sequence exhibited no significant similarity to human proteins, supporting its predicted immunological safety. Collectively, this rationally designed construct is expected to activate both humoral and cell-mediated immune responses in a synergistic manner, highlighting its potential as a vaccine candidate against *C. violaceum* (Figure 3).

### 2.6. Post-Translational and Physicochemical Characterization of the Vaccine Construct

Physicochemical and immunological properties of the final multi-epitope vaccine (MEV) construct were examined extensively to confirm the appropriateness of the construct in terms of expression, stability, and immunogenicity. Research was conducted in the ProtParam server that provided data about the significant parameters, which are relevant to the recombinant production and biological activity. The construct with a calculated molecular weight of approximately 28.52 kDa is an optimal size to be expressible in a heterologous system and purified after the expression. The theoretical isoelectric point (pI) was estimated at 9.62 indicating weakly basic nature that has the potential to influence the solubility and electrostatic interactions under physiological temperatures. The results of charge distribution analysis revealed 27 basic and 12 acidic residues (Lys, Arg and Asp, Glu), which is in line with the electrostatic profile that is balanced and which has proven to add to structural stability and solubility. The instability index assessment gave a score of 27.12, which is stable, and can be stored and used in future experimental applications. In addition, an aliphatic index of 66.43 was the indication of improved thermostability on the basis of the capability of retaining the conformational stability on a wide temperature scale. The GRAVY of −0.179 corresponded to a hydrophilic nature and desired solubility in aqueous biological factors and thus enhanced interactions with the immune system of the host. In vivo half-life computed only contributed to construct stability in hosts: in mammalian reticulocytes, more than 30 h and, in yeast, more than 20 h and more than 10 h in *E. coli*. These emanate out of its outlook in terms of strong expression across systems. To analyze the constructs in terms of safety and immunogenicity potential, VaxiJen v2.0, AllerTOP v2.0, and ToxinPred were used to predict the construct. The results proved that the MEV is antigenic, non-allergenic, and non-toxic and, therefore, a potential candidate to be used as a vaccine. In general, the evaluation of stability of the structure, thermostability, solubility, and desired immune properties is highly favorable to the MEV construct as a potential candidate to be further tested in preclinical studies and approved as a subunit vaccine.

**Figure 4:**
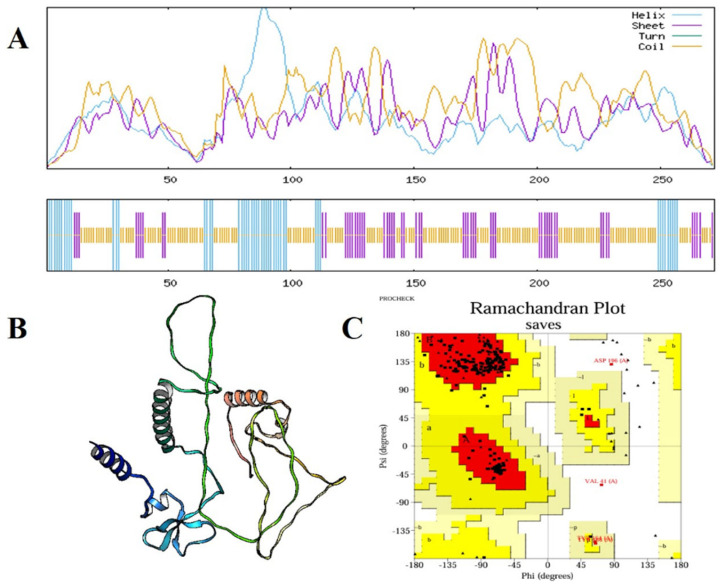
Structural prediction and validation of the multi-epitope vaccine (MEV) construct. (**A**) SOPMA secondary structure analysis of the MEV construct, representing helix (cyan), sheet (purple), and random coils (yellow) distributed along the protein sequence. (**B**) Optimized tertiary structure of the vaccine construct created and optimized by computational modeling, representing its overall 3D conformation. (**C**) Ramachandran plot confirmation of the predicted structure, with 92.1% of the residues found in the most favorable locations, 5.8% in further allowed locations, and merely 1.1% in disallowed locations, endorsing the stereochemical quality and stability of the construct.

**Figure 5:**
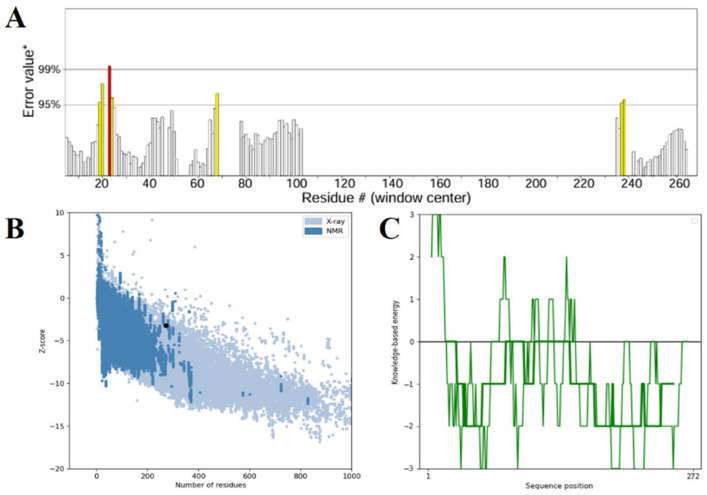
(**A**) Structural confirmation of the ERRAT server-validated refined 3D vaccine model. The plot demonstrates the overall quality factor against residue positions, where areas of the model rejected at the 99% confidence level are in red and those rejected at the 95% confidence level are in yellow. (**B**) The ProSA-web Z-score plot of the improved model (indicated by a black dot) against the Z-scores of experimentally determined protein structures solved using X-ray crystallography (light blue) and NMR spectroscopy (dark blue), indicating the quality of the model overall. (**C**) The ProSA-web energy plot depicting residue-wise knowledge-based energy values, where negative values denote energetically favorable areas of the structure, affirming the validity of the modeled vaccine construct.

The secondary and tertiary structural analysis was performed to holistically assess the structural organization of the developed multi-epitope vaccine (MEV). The protein of interest was a predicted 272 amino acid construct called MEV that has a predicted secondary structure composition as shown by the SOPMA server: 165 random coils (60.66%), 56 extended 20.59% of total 272 amino acid 51 56 extended 20.59% of total 272 amino acid 51 56 extended 20.59% of total 272 amino acid 51 56 extended 20.59 Such a balanced pattern of structural motifs is indicative of a well-folded and stable protein conformation that is a condition of functional integrity and efficient immune system recognition of the antigen. Due to the AlphaFold server, a deep-learning-based 3D protein structure prediction server, the 3D structure of the tertiary structure was expected. Stereochemical quality improvement was then achieved further by refining the original structure using the GalaxyRefine platform, which repacks side chains and minimizes the entire energy of the model to enhance global and local model accuracy. It was also confirmed with the help of Ramachandran plot analysis that 92.1 percent of the residues were in the most preferred regions of the conformation, 5.8 percent were in the additional allowed regions and only 1.1 percent were in the disallowed regions. Large percentage of residues in preferred areas indicate that the predicted structure is stereochemically viable (Figure 4). Other structural quality checks were made with ProSA-web (accessed on 11 April 2025) and ERRAT (accessed on 12 April 2025). The quality factor was found to be 93.519 in the ERRAT check that attests to the correct high degree of structure. At the same time, the ProSA Z-score of −3.2 fell far within the range of the generally expected proteins of similar length determined by an experiment and therefore, ruled out the possibility of any serious structural defects or misfolded regions (Figure 5). Taken together, these data demonstrate that the optimized MEV construct has a well-folded, stable and functionally relevant tertiary structure which also makes it a viable candidate to follow-up experimental testing and even to develop a vaccine.

### 2.7. Structural Conformity Analysis of B-Cell Epitopes

To validate the structural fidelity of the selected B-cell epitopes, we additionally modeled the native parent proteins Pbp1A and LptD using AlphaFold2. The two epitopes DLAGKTGTTSDWKDAW (Pbp1A) and RVKAGDRFRMTRGGDV (LptD) were located and extracted from their respective native structures. The same epitope regions were also isolated from the MEV construct. Structural alignments were performed using PyMOL v.1.3 (super algorithm) and TM-align. The results demonstrated strong conformational similarity between the native epitopes and their MEV-embedded counterparts. The RMSD values were 1.62 Å for DLAGKTGTTSDWKDAW and 1.48 Å for RVKAGDRFRMTRGGDV, indicating excellent structural preservation. These results confirm that integration of the epitopes into the MEV construct did not distort their native antigenic geometry.

### 2.8. Prediction and Selection of B-Cell Epitopes

**Figure 6:**
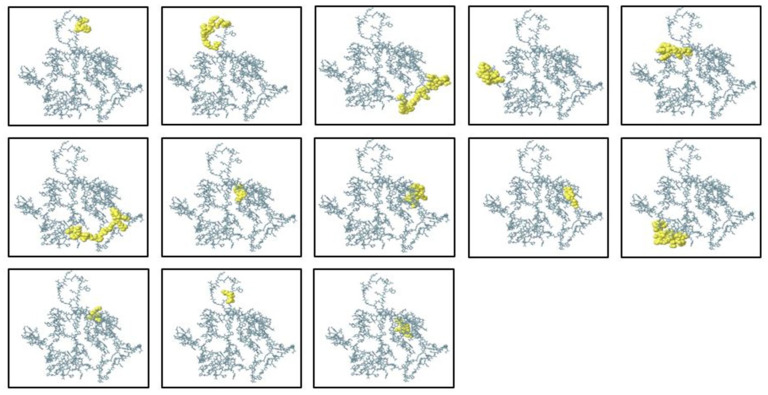
Predicted discontinuous (conformational) B-cell epitopes of the ElliPro server mapped onto 3D structure of the multi-epitope vaccine construct. The epitope residues are colored yellow, while the rest of the protein structure is in grey, illustrating the spatial distribution of antigenic sites that may be involved in antibody recognition.

B lymphocytes (B cells) are a key part of the humoral arm of adaptive immunity, working to neutralize infectious pathogens through the synthesis of target-specific antibodies. Therefore, the inclusion of optimal B-cell epitopes is an essential step in the construction of subunit vaccines, since they induce strong and specific antibody-based responses. In the current research, both conformational (discontinuous) and linear (continuous) B-cell epitopes were predicted by the ElliPro server, which uses the three-dimensional structural data of the protein to detect surface-exposed and antibody-accessible sites. The completed vaccine construct in its 3D structure was input into ElliPro, and epitope prediction was performed under default settings, using a minimum score cut-off of 0.5 and a maximal distance cut-off of 6 Å. These parameters made it possible to identify epitopes with ideal accessibility and structural flexibility. The prediction resulted in 13 conformational epitopes of 3 to 29 residues in size, scoring between 0.576 and 0.906, indicating that they were of high potential to be involved in immune recognition. Concurrently, 9 linear epitopes were discovered, expanding the antigenic map of the construct and enhancing its ability to induce humoral immunity. To confirm their spatial distribution and determine surface accessibility, the mapped epitopes were visualized with PyMOL v1.3 (Figure 6). The concerted occurrence of linear and conformational B-cell epitopes in the construct should augment both the breadth and extent of the immune response and thereby maximize the predicted immunogenic potential of the multi-epitope vaccine candidate.

### 2.9. Molecular Docking Analysis with Host Immune Receptor

**Figure 7:**
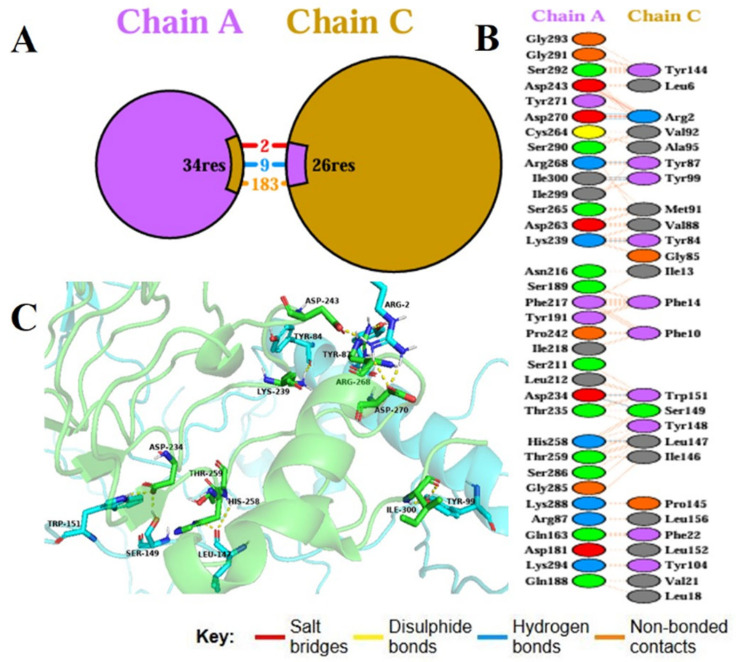
Chain A–Chain C molecular docking interaction. (**A**) Overall binding interface between Chain A and Chain C, indicating the number of interacting residues contributed by each chain, with colored segments corresponding to residues forming salt bridges (red), hydrogen bonds (blue), and non-bonded contacts (orange). (**B**) List of individual interface residues from Chain A and Chain C, with each residue pair color-coded according to interaction type: red for salt bridges, blue for hydrogen bonds, and grey for non-bonded contacts; no disulphide bonds were detected and therefore yellow is omitted from the legend. (**C**) Three-dimensional view of the docking complex highlighting key interface residues from both chains and depicting salt bridges (red lines), hydrogen bonds (blue lines), and non-bonded contacts (grey dashed lines) that contribute to the stability of the complex.

Molecular docking is a basic computational method applied to study binding affinity, interaction stability, and interfacial kinetics of vaccine constructs with host immune receptors, thus revealing their immunological potential. In the current work, the binding between the constructed multi-epitope vaccine (MEV) and Toll-like receptor 4 (TLR4), an important pattern recognition receptor playing a central role in innate immune defense, was studied with the ClusPro server, an already established protein–protein docking tool. The optimized 3D structure of the MEV was used as the ligand, and the human TLR4 crystal structure (PDB ID: 2Z62) was utilized as the receptor. Ten potential docking conformations were predicted by ClusPro, and the top-ranked cluster included 96 members with the least interaction energy of −1336.4 kcal/mol, reflecting a very stable vaccine–receptor complex. To better describe molecular contacts, the PDBsum server was used, which indicated that the MEV established 9 hydrogen bonds with TLR4 Chain A, further defining the structural compatibility and specificity of the complex (Figure 7). Thermodynamic viability was then determined using the PRODIGY server and was estimated at a Gibbs free energy (ΔG) of −16.2 kcal/mol. This translated to a dissociation constant (Kd) of 1.3 × 10^−12^ M at 37 °C, which validated the strong and favorable binding affinity. Collectively, these docking results demonstrate that the MEV construct forms a stable, energetically favorable, and specific interaction with TLR4. This strong binding affinity speaks volumes for its potential to activate innate immune signaling cascades, thereby triggering defensive immune reactions upon inoculation.

### 2.10. Normal Mode Analysis of the MEV–TLR4 Complex

**Figure 8:**
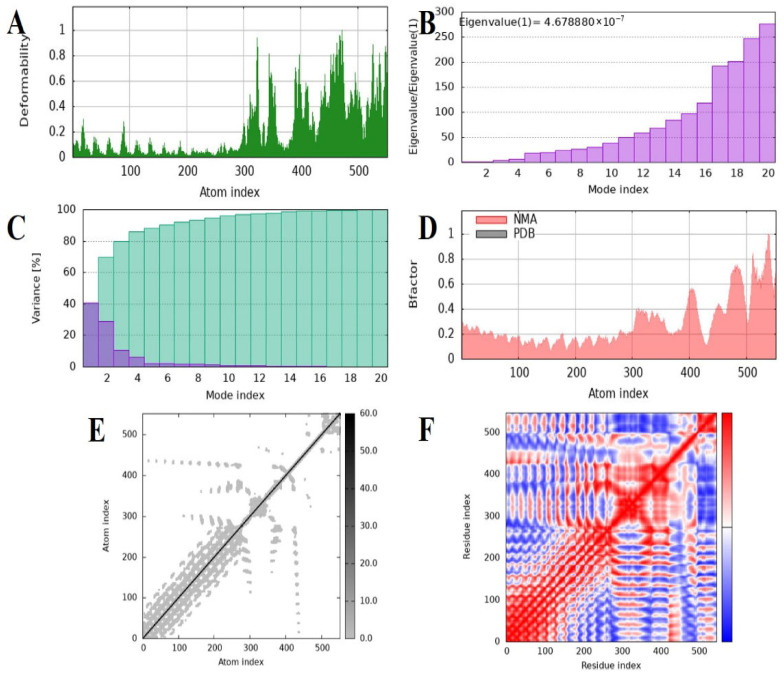
Normal mode analysis and molecular dynamics characterization of docked vaccine–receptor complex carried out with iMODS. (**A**) Deformability plot of the calculated flexibility per atom, where peaks represent areas of greater structural mobility. (**B**) Eigenvalue distribution normalized by the first mode eigenvalue over the first 20 modes, showing relative stiffness; lower eigenvalues correspond to greater conformational flexibility. (**C**) Each normal mode’s variance explained as a percentage, demonstrating that low-frequency modes account for the majority of the motion overall. (**D**) Comparison of B-factors for normal mode analysis (NMA, red shaded) and experimentally reported B-factors, demonstrating atomic displacement consensus. (**E**) Atomic motion covariance matrix heatmap, where correlated motions are represented in darker colors and anti-correlated motions are in blue, indicating dynamic coupling between residue pairs. (**F**) Elastic network representation of interatomic interactions; dots represent springs that connect the atoms, and darker intensities represent stronger elastic forces stabilizing the protein structure.

Dynamic behavior, conformational flexibility, and intrinsic stability of the MEV–TLR4 complex were also investigated by normal mode analysis (NMA) by using the iMODS server. This technique evaluates collective movement in biomolecular complexes by utilizing internal coordinate mechanics, and therefore provides data on their deformability along with structural stability. The profile of deformability revealed constrained flexibility across the majority of residues, suggesting a strongly rigid complex with minimal conformational deformation (Figure 8A). As expected, B-factor predictions from NMA revealed smaller deviations than the experimentally obtained B-factors, which suggested reduced atomic fluctuations and confirmed the limited mobility of the complex (Figure 8B). The principal mode eigenvalue was computed as 4.67888 × 10^−7^, which reflects a high rigidity of the complex as well as a considerable energy demand for the perturbation of structure. Progressive eigenvalue increases with higher modes suggested uniform vibrational stiffness, complementing the system’s robustness (Figure 8C). The analysis of variance revealed a drop in the contribution of single modes, confirming well-organized inter-residue motions and low conformational entropy (Figure 8D). Just like the covariance matrix projected the correlations of residue motions onto a map and revealed patterns of positively correlated (red), negatively correlated (blue), and uncorrelated (white) motions, presenting a complete picture of the internal dynamics (Figure 8E). An elastic network model was also created to demonstrate the mechanical robustness of interatomic interactions. Darker gray points indicated stronger and more rigid connections in this representation, with spring-like connectors representing residue–residue interactions (Figure 8F). On the whole, the NMA results validated that the MEV–TLR4 complex exhibits high structural stability, decreased deformability, and pronounced internal rigidity, all cumulatively favoring the thermodynamic stability and structural persistence of the vaccine–receptor complex under dynamic conditions.

### 2.11. Molecular Dynamics Simulation of the Protein Complex

**Figure 9:**
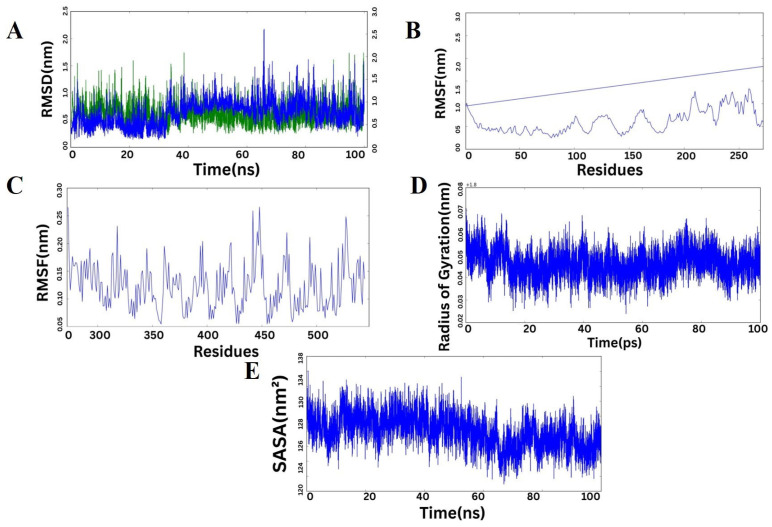
Molecular dynamics simulation analysis of the protein complex. (**A**) RMSD trajectory of Chain A (green) and Chain C (blue) over 100 ns, showing stable conformations with minimal structural deviation. (**B**) RMSF plot for Chain C illustrating residue-based flexibility, with greater fluctuations found mainly at loop and end segments. (**C**) RMSF plot for Chain A revealing mostly rigid residues with only minor flex sites. (**D**) Plot of radius of gyration (Rg) versus simulation time, showing the preserved compactness of the complex. (**E**) Solvent-accessible surface area (SASA) fluctuations reflecting uniform solvent exposure and stable surface packing throughout the simulation. Overall, the data collectively suggest that the complex is structurally stable and dynamically favorable throughout the MD simulation.

The dynamical stability and behavior of the protein complex formed by Chain A and Chain C were assessed using various MD simulation parameters (Figure 9). The RMS-D profiles (Figure 9A) of both Chain A and Chain C showed no appreciable structural drifts during the 100 ns simulation, confirming that the two chains were structurally stable. Chain A fluctuated predominantly between ~0.5 and 1.5 nm, and Chain C fluctuated around ~0.5 to 2.0 nm, indicating that both chains were in equilibrium states with negligible conformational alterations. RMSF profile analyses of Chain A (Figure 9C) and Chain C (Figure 9B) indicated most residues contained limited flexibility, with higher peaks being predominantly confined at loop and terminus regions that are inherently mobile and necessary for molecular recognition. The central residues remained nearly stationary, favoring persistent interchain contacts and complex stability. The radius of gyration (Figure 9D) was fairly constant with only slight transient variations, suggesting maintenance of the overall compactness and lack of any extreme unfolding events over the course of the simulation. The solvent-accessible surface area (SASA) values (Figure 9E) also indicated slight variations across the trajectory, suggesting a stable solvent exposure and implying the complex had a closely packed interface. When considered collectively, these MD simulation parameters all together illustrate that the protein complex retains structural integrity, dynamic stability, and compactness during the simulation period, in favor of its potential biological function.

### 2.12. Immune Simulation Analysis

**Figure 10:**
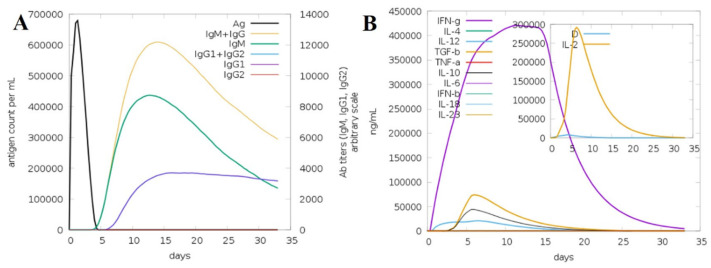
In silico immune simulation of the multi-epitope vaccine (MEV) over 35 days. (**A**) Antigen levels (black) decline rapidly after immunization, while IgM, IgG1, and IgG2 antibody responses rise, reflecting strong primary and memory humoral immunity. (**B**) Cytokine profiles show a prominent IFN-γ response and transient IL-2 peak (inset), alongside moderate levels of other cytokines, indicating balanced cellular immune activation. These results highlight the vaccine’s ability to elicit comprehensive and durable immune protection.

The immunogenic potential of the engineered multi-epitope vaccine (MEV) was evaluated in silico using the C-ImmSim platform to simulate immune responses over a 35-day period. The simulation demonstrated that the MEV induced a robust and multi-faceted immune response involving both humoral and cellular immunity (Figure 10). Panel A displays the antigen clearance kinetics alongside antibody titers. A sharp initial peak in antigen count was observed immediately after administration, which rapidly declined by day 5, signifying effective antigen neutralization by the immune system. The primary immune response started with a significant rise in IgM titers, indicative of early antigen recognition. Subsequent booster doses triggered strong secondary and tertiary immune responses, marked by prominent increases in IgG1 and IgG2 subclasses, as well as a cumulative IgM + IgG response, demonstrating effective antibody class switching and maturation. Notably, IgG1 and IgG2 levels remained elevated through the later days, suggesting the establishment of persistent humoral immunity. Panel B illustrates cytokine and interleukin profiles post-immunization. A substantial upregulation of IFN-γ was observed, peaking around day 15, indicating activation of T-helper 1 (Th1) responses critical for cell-mediated immunity. IL-2 showed a sharp but transient peak early during the response, supporting T cell proliferation. Moderate expression of IL-4, IL-6, IL-10, IL-12, IL-18, IL-23, TGF-β, TNF-α, IFN-β, and IL-1β was observed throughout, reflecting a balanced cytokine milieu that promotes immunogenicity while minimizing excessive inflammatory reactions. The inset calls attention to the IL-2 transient kinetics, which underline early immune activation. In total, these findings verify that the MEV construct sufficiently activates initial innate responses and induces strong adaptive humoral and cellular immunity, with proper antibody class switching and balanced cytokine milieu. The increasing antigen clearance and long-lasting antibody titers demonstrate the vaccine’s ability to induce sustained protection. This in-depth immune profile substantiates the MEV as a potential vaccine candidate warranting further experimental confirmation.

### 2.13. Codon Optimization and In Silico Cloning

**Figure 11:**
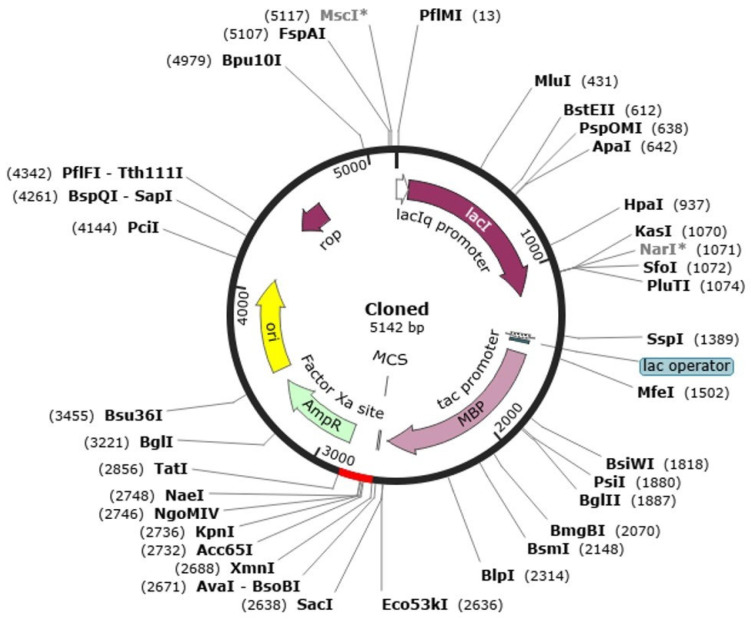
Representation of the in silico cloning of the designed vaccine construct into the *E. coli* K12 expression system. The plasmid backbone is illustrated in black, whereas the integrated nucleotide sequence corresponding to the vaccine insert is displayed in red.

A codon optimization and in vitro cloning are necessary to maximize the efficient expression of the heterologous host Escherichia coli. This study used the Java Codon Adaptation Tool (JCat) to optimize the codon usage of the designed multi-epitope vaccine (MEV) to the *E. coli* K12 preferences of its expression. The optimized nucleotide sequence was that of 272 amino acid sequences of the MEV protein. The optimized sequence had Codon Adaptation Index (CAI) of 1.0, which is the range of optimality (0.8 to 1.0), and GC content of 57.84, also within the range of optimality of 30–70. Combination of these measures suggests that there is great efficiency in the bacterial system in transcription and translation. Upon optimization, the optimized gene of the vaccine was in silico cloned into pET-28a(+) expression vector, which is highly utilized to express recombinant proteins. The resulting recombinant plasmid construct was in total 5142 base pairs in length, which made it easy to integrate the vaccine gene and made it viable in further use like large scale expression and purification of the gene in *E. coli*. This move gives a nice platform of experimental manufacturing and validation of the MEV construct (Figure 11).

## 3. Discussion

*Chromobacterium violaceum* leads to many complications in immunocompromised individuals, including, meningitis, puerperal sepsis, urinary tract infections, orbital cellulitis, chronic cellulitis, septic spondylitis, diarrhoea, osteomyelitis retropharyngeal infection, endocarditis, internal jugular vein thrombophlebitis brain abscess, hemophagocytic syndrome neutropenic sepsis and chronic granulomatosis [40]. *C*. *violaceum* possesses inherent resistance to many first-line antibiotics, particularly beta-lactam antibiotics and penicillins [41]. Additionally, it is also resistance to some second line antibiotics including amoxicillin [9]. Healthcare professionals are actively evaluating alternative therapeutic approaches, such as combinatorial regimens and next-generation antimicrobial agents, to mitigate these clinical challenges. There are several methods being used to treat bacterial infection such as antibiotic therapy, antimicrobial peptides, immune therapy and vaccines. Although, vaccines are widely used for the treatment of bacterial infections and it saved millions of people of deadly infections [42]. Conventional vaccine development methodologies are characterized by substantial financial costs, extended timelines and significant labor demands, while also being associated with a high likelihood of attrition across successive experimental phases. Such computational methodologies have demonstrated their effectiveness against a range of pathogens, including *Mycobacterium tuberculosis*, *Klebsiella pneumoniae*, *Ruminococcus gnavus* and *Leptospira interrogans* [21,22,43,44]. Furthermore, recent evidence demonstrating that MEVs predicted through reverse vaccinology can indeed be validated successfully in vivo and in vitro [43,45,46,47].

Pan-genome includes the entire genetic collection of all the strains of a particular species, including the conserved core genome [48]. Whereas, the core genome plays a pivotal role in conserving essential cellular functions and also in bacterial evolution. The accessory genome is the variable portion of the pan genome that contributes to pathogenicity, antibiotic resistance and stress tolerance [49]. In our analysis, the pan-genome of *C*. *violaceum* was constructed using all eight genomes available in GenBank database. I used the PanExplorer platform for this purpose and identified 3144 core genes conserved across all strains. The reason why the core genome was targeted is its evolutionary conservation and stability within all strains, which increases the chances of finding essential and broad-spectrum therapeutic or vaccine targets. This strategy concurs with earlier pangenomic studies in which core genes were targeted for discovery [50,51]. First of all, the gene sequences were translated into respective proteins than essential genes were screened using the Geptop 2.0 server. I identified 360 proteins that were essential for bacterial survival. After that I removed 239 human homologs by using BlastP with E-value cutoff ≤ 10^−4^ and sequence identity < 35% and it results in 121 proteins. The removal of human homologous proteins minimizes the potential for cross-reactivity during therapeutic treatments against the bacteria [52]. Finally, our computational analysis screened two essentials proteins, Organic solvent tolerance protein (LptD), a key player in LPS assembly and penicillin-binding protein 1A (Pbp1A) which could be best potential vaccine candidates. Lipopolysaccharides (LPS) play critical roles in protecting bacteria against adverse environmental stresses and harmful agents, including antibiotics [53]. Among the proteins involved in LPS biogenesis, LptD is particularly notable, as it assembles into a unique 26-stranded β-barrel structure [54]. To date, this β-barrel represents the most extensively characterized structure of its kind [55]. Elucidating the structural features of LptD provides valuable insights into its functional role in LPS assembly and underscores its importance for bacterial viability [56]. PBP1A plays a pivotal role in the terminal stages of bacterial cell wall biosynthesis by cross-linking peptidoglycan chains, the fundamental structural components of the cell wall [57]. The bacterial cell wall not only provides structural support and maintains cellular morphology but also safeguards the cell against osmotic stress [58]. Consequently, bacterial viability is critically dependent on the proper functioning of PBP1A during peptidoglycan assembly [59]. Importantly, PBP1A is a well-established target of penicillin and other β-lactam antibiotics [60]. However, resistance frequently emerges through multiple mechanisms, including the production of β-lactamases that degrade β-lactams, as well as structural modifications in PBPs that reduce antibiotic affinity [61]. Mutations within PBP1A itself can also confer intrinsic resistance to β-lactam antibiotics, allowing bacterial survival under antibiotic pressure [62]. These resistance mechanisms underscore the therapeutic significance of PBP1A, not only as a classical drug target but also as a promising candidate for structure-guided drug design and immunoinformatics-driven vaccine strategies. Targeting conserved regions of PBP1A could help circumvent resistance-associated mutations and contribute to the development of next-generation therapeutics against drug-resistant bacterial pathogens [63]. To avoid the risks associated with autoimmune reactions rigorous antigenicity and allergenicity evaluation were also performed by using VaxiJen v2.0 with a cut-off of 0.5 and AllerTOP v2.0, respectively [21]. A rationally designed combination of immunodominant epitopes has the potential to induce effective immune response compared to the native antigen [27]. Rigorous screening yielded epitopes predicted to elicit both humoral and cellular immunity. Therefore, the identification of epitopes capable of efficiently eliciting an immune response remains a major challenge in vaccine development [64]. Human β-defensins, including hBD-4, are known to function as natural antimicrobial peptides and TLR agonists, providing a rational basis for their selection as adjuvants in the vaccine construct. Here i designed a multi epitope vaccine (MEV) by incorporating highly antigenic B-cell and T-cell epitopes and i further validated our results by population coverage analysis, which predicted an 89% global applicability [65].

The 272-amino-acid MEV construct was rationally designed and optimized using peptide linkers such as GPGPG, AAY, and KK to ensure appropriate epitope spacing, efficient antigen processing, and preservation of epitope integrity, thereby enhancing the overall immunogenic potential of the construct [66,67]. Adjuvants played a crucial role in augmenting the immunogenicity of the construct by stimulating innate immune responses and facilitating antigen presentation [68]. Physicochemical evaluation using ProtParam revealed a molecular weight of approximately 28.52 kDa, an antigenicity score of 1.5906—indicative of strong immunogenic potential—and a solubility score of −0.254, suggestive of moderate solubility. Structural characterization through SOPMA and AlphaFold, followed by refinement using GalaxyRefine, predicted reliable secondary and tertiary structures. Structural superimposition of the epitopes in their native proteins and within the MEV construct yielded RMSD values below 2 Å, confirming that the epitopes retained their native conformations. This preservation is essential for enabling appropriate B-cell recognition and supports the immunogenic relevance of the selected epitopes. The structural validation analyses confirmed the overall conformational stability and immunological soundness of the designed MEV construct. Model validation with ProSA-web yielded a Z-score of −3.2, confirming acceptable structural quality and reliability [69]. Codon optimization of the construct further supported its suitability for expression in Escherichia coli, with a Codon Adaptation Index (CAI) of 1.0, within the optimal range (0.8–1.0), and a GC content of 57.84%, also within the optimal window of 30–70%. Additionally, intrinsic disorder analysis indicated that the MEV construct contains only minor flexible regions, suggesting overall structural organization [70]. The incorporation of EAAAK and GPGPG linkers is known to support controlled domain separation and antigen processing, while KK linkers were used to maintain epitope independence without adversely affecting the predicted global stability of the construct [71].

Molecular docking and dynamics simulations demonstrated strong and stable interactions between the MEV and immune receptors, particularly Toll-like receptor 4 (TLR4), underscoring the potential of the construct to activate innate immune signaling [72]. This was further supported by immune simulation analyses, which predicted robust primary and secondary immune responses, along with the capacity to elicit long-term protective immunity [73,74]. Although molecular docking and dynamics simulations provide valuable predictive insights, these results remain theoretical and require experimental validation. Future research will include in vitro and in vivo assays to confirm receptor binding, immune responses, and protective efficacy of the designed MEV construct [75]. Previous studies have employed standard reverse vaccinology pipelines for vaccine design; however, our study distinguishes itself through the integration of a species-wide pangenome analysis to prioritize core antigens conserved across all circulating strains of *C. violaceum*. This ensures broader coverage and higher translational relevance. Furthermore, our use of human β-defensin-4A as an adjuvant provides a physiologically relevant, host-compatible molecule to potentiate immune responses, which is rarely explored in previous computational vaccine design studies. Taken together, the development of this construct against *C. violaceum* represents a promising advance toward novel therapeutic strategies, particularly in combating the growing threat of antibiotic resistance and emerging bacterial pathogens.

## 4. Materials and Methods

### 4.1. Genome Retrieval and Dataset Preparation for Pangenome Analysis

The whole-genome assemblies of eight strains of *C. violaceum*, namely ATCC 12472, CV1192, CV1197, CV20, FDAARGOS_1273, FDAARGOS_1274, FDAARGOS_635, and NCTC9695, were downloaded from the NCBI GenBank database [76]. The assemblies were selected to be representative of the genomic diversity of the species and included strains from various geographical locations and ecological niches [77]. Pangenome analysis was conducted with the PanExplorer web server https://panexplorer.southgreen.fr (accessed on 15 June 2025), which combines multiple cutting-edge tools for comparative genomics [78]. For this analysis, the Roary pipeline was utilized in PanExplorer for clustering orthologous coding sequences among the genomes that had been chosen. Roary uses an all-against-all BLASTP similarity search with subsequent application of the Markov Cluster Algorithm (MCL) for classification of homologous proteins into orthologous groups [51]. An 80% minimum BLASTP sequence identity threshold was employed for orthology classification, whereas all other settings were set at their defaults. The resulting gene clusters were divided into three classes: core genes (common to all genomes), dispensable or accessory genes (shared in two or more but not all genomes), and strain-specific genes (exclusive to one genome). Pangenome visualization charts were generated using the Highcharts web-based plotting library https://www.highcharts.com (accessed on 15 June 2025). Of these, the core genome was used for downstream subtractive proteomics [79].

### 4.2. Subtractive Proteomics Analysis

The core gene set derived from the pangenome analysis was then translated to protein sequences with the use of the bacterial genetic code in Biopython. Complete open reading frames only were kept, and truncated sequences were removed to guarantee precise downstream analyses [80,81]. Only proteins encoded within the conserved core genome were considered for downstream analysis to ensure cross-strain vaccine coverage. Plasmid-borne proteins were excluded because of their variable presence and limited evolutionary stability among *C. violaceum* isolates. The resulting translated core proteome was then subjected to a subtractive proteomics pipeline to determine possible therapeutic and vaccine candidates [82]. First, the Geptop 2.0 server was used to identify essential proteins based on orthology and evolutionary characteristics [83,84]. Proteins that were predicted as essential were regarded as being required for bacterial fitness and survival and were considered prime initial candidates. For removing host homologs and reducing the likelihood of cross-reactivity with human proteins, the essential protein list was analyzed using a BLASTp search against the human reference proteome (taxid: 9606). Proteins with more than 30% identity and E-value ≤ 1 × 10^−4^ with human proteins were rejected (Barh et al., 2011; Gupta et al., 2013) [38,39]. Non-homologous proteins alone were considered for further prioritization. Subcellular localization of the non-host homologous proteins was predicted with the help of the PSORTb v3.0 server [85]. Proteins that were predicted to localize in the outer membrane, extracellular space, or periplasm were given priority since these topologies make them more accessible to the host immune system and more amenable as vaccine targets. The transmembrane topology of shortlisted proteins was then evaluated with TMHMM v2.0 [86]. Proteins with more than one transmembrane helix were not considered, as they are typically tricky to express and isolate experimentally. Only those proteins that had zero or one predicted helix were kept. Lastly, the antigenicity of the potential proteins was assessed by the VaxiJen v2.0 server [86,87]. The antigenicity of the proteins was set at a threshold of 0.5 and the proteins with a value of antigenicity above this were considered to be likely antigens. These were the non-host, antigenic, surface-exposed proteins that were believed to be the most promising targets of downstream epitope mapping and vaccine design.

### 4.3. Epitope Prediction and Screening

#### 4.3.1. CTL Epitope Selection and Assessment

On the Immune Epitope Database (IEDB), the MHC-I binding tool was used to predict cytotoxic T lymphocyte (CTL) epitopes of the shortlisted proteins in the vaccine candidate proteins [88]. Consensus prediction strategy was employed, which is a combination of a few algorithms that optimize it in order to predict epitopes with high binding affinity in a broad pool of HLA alleles. The selection of the epitopes was further made based on consensus score of below 2.0 (Kim et al., 2012) [89]. Immunogenicity of the shortlisted CTL epitopes was next evaluated through IEDB immunogenicity prediction tool which predicts immunogenicity of the epitopes to cause potent T-cell mediated immune response (Calis et al., 2013) [90]. Moreover, it was determined that the antigenicity of each of the epitopes was measured by the VaxiJen v2.0 server using the default threshold of 0.5 so as to incorporate the epitopes that had high immunogenic profiles (Doytchinova and Flower, 2007) [91]. To be on the safe side, an allergenic and toxic risk of all CTL epitopes was determined with the AllerTOP v2.0 server and ToxinPred server, respectively [39,92]. Only the epitopes that had been predicted to be antigenic, non-allergic and non-toxic were retained and incorporated into the vaccine construct.

#### 4.3.2. HTL Epitope Selection and Analysis

The helper T lymphocyte (HTL) epitopes prediction was performed on the IEDB resource MHC-II binding predictor (Vita et al., 2019) [88]. Only 15-mer epitopes were predicted and it has been known that this size of epitope is detected by HLA-DR alleles, thus covering the maximum population [93]. The epitopes were chosen on the basis of the criteria of high binding affinity, antigenicity and immunogenicity. VaxiJen v2.0 was also used to test the antigenicity of the selected HTL epitopes, AllerTOP v2.0 tested the allergenicity of the selected HTL epitopes and ToxinPred tested toxicity of the selected HTL epitopes [91,92]. It was done in such a stringent manner that highly immunologically significant epitopes would be selected, as well as to guarantee safety and suitability to be incorporated in the multi-epitope vaccine construct.

#### 4.3.3. LBL Epitope Identification and Analysis

ABCpred server, a machine learning system predicting protein sequence B-cell epitopes, also predicted linear B-cell lymphocyte (LBL) epitopes [94]. The cut-off point was determined as 0.5 therefore providing guaranteed identification of epitopes that had high binding probabilities. The selected epitopes have also been investigated based on the antigenicity (VaxiJen v2.0), allergency (AllerTOP v2.0) and toxicity (ToxinPred) [39,91,92]. Only those epitopes that were predicted to be antigenic, non-allergenic and non-toxic were conserved. To exclude any potential autoimmune cross-reactivity, each shortlisted CTL, HTL, and B-cell epitope was screened against the human proteome (taxid: 9606) using BLASTp short-sequence analysis. Epitopes exhibiting >30% identity or significant alignment (E-value ≤ 1 × 10^−4^) were removed. Because the MEV construct consists entirely of these non-homologous epitopes, the full 272-amino-acid vaccine sequence also inherently shows no relevant similarity to human proteins.

### 4.4. Population Coverage Analysis

To study the worldwide coverage of the predicted epitopes, the population coverage was completed using the Immune Epitope Database (IEDB) population coverage tool [95]. This tool is formed by the combination of the data on allele frequencies and estimates of the potential of epitope recognition in different human populations. The analysis was based on HLA allele distribution data of 11 major geographical regions and 78 distinct ethnic groups in the world, therefore making it easy to establish the breadth of immunogenicity to the selected epitopes [96]. By considering the polymorphic characteristics of HLA alleles, this analysis will provide that the prepared vaccine construct is well-covering the entire population, making chances of population-specific immune escape unlikely.

### 4.5. Designing of the Vaccine Construct

Multi-epitope vaccine (MEV) construct was developed by the combination of LBL, CTL, and HTL epitopes, with the help of the suitable adjuvant. Adjuvants play an important role in enhancing vaccine construct immunogenicity through the induction of innate immune responses and antigen presentation. The adjuvant adopted in this study was human 2-defensin-4A (HBD-2, NCBI Accession No: NP 004933.1). 2-defensins are small, cationic antimicrobial peptides which are agonists of TLR4, thereby initiating innate immunity and mediating the interaction between innate and adaptive immunity [97,98]. Human β-defensin-4A (hBD-4) is reported to activate Toll-like receptor 4 (TLR4) and enhance antigen presentation, thereby exhibiting intrinsic adjuvant activity [99,100]. In order to achieve structural flexibility and the best presentation of epitopes, some linkers were used. The adjuvant was coupled to the CTL epitopes with EAAAK, and this process confers rigidity, functional separation between the domains. The AAY linkers were applied to couple the CTL epitopes to undergo proteasomal degradation and presentation on the MHC-I molecules [101]. Similarly, the good presentation of HTL epitopes on MHC-II molecules was achieved through the use of GPGPG linkers between the epitopes. For linear B-cell epitopes (LBLs), bi-lysine (KK) linkers were incorporated to maintain epitope independence and prevent functional epitope formation rather than to confer proteolytic stability. Importantly, the overall stability of the construct was evaluated at the full-length protein level, confirming that inclusion of KK linkers did not adversely affect structural integrity or safety [102]. To exclude the possibility that linker integration generated novel human-like motifs, the complete MEV construct, including the adjuvant and all linker sequences, was screened against the human proteome (taxid: 9606) using BLASTp, confirming the absence of any significant sequence similarity [103]. The final MEV construct is thus a possible vaccine against *C. violaceum*.

### 4.6. Structural Analysis

In order to investigate the structural properties of the multi-epitope vaccine (MEV) construct in a systematic manner, the diversity of bioinformatics tools was used to identify the stability, functionality, and the potential of the vaccine of the construct. Theoretical isoelectric point (pI), molecular weight (MW), instability index (II), aliphatic index (AI), overall average of hydropathicity (GRAVY), and half-life at in vivo and in vitro were calculated on ProtParam server [104]. These parameters provide indications on the nature of stability and solubility of the construct to be designed generally. The antigenic and immunogenic potential of the MEV construct to ascertain that it can induce good immune responses was determined by the VaxiJen v2.0 server and the IEDB tools of immunogenicity prediction [87,88]. To confirm the safety, the ability of the vaccine candidate to cause an allergy was deduced on the AllerTOP v2.0 server [92]. The SOPMA tool has been used in establishing the percentage number of 3-helixes, 3-turns, protruded strands and arbitrary coils of the vaccine construct in the case of secondary structure prediction [105].

### 4.7. Refinement, Confirmation and Prediction of Tertiary Structure

Proper prediction of tertiary structure of MEV construct is important to determine its stability, folding and functional significance. To predict the first three-dimensional structure of the MEV construct, the AlphaFold server highly accurate deep-learning-based protein structure modeling platform was used and then the structure was refined structurally using the GalaxyRefine server which refines local structural conformations and minimizes side-chain packing to optimize the stereochemical quality of the model [106]. The refinement was aided by structural quality and stereochemical parameters of the resulting model that were verified with assistance of the RAMPAGE server which measures the geometry of the backbone in terms of Ramachandran plots [107]. Structural quality and stereochemical parameters of the resulting model were verified after the refinement with the help of the RAMPAGE server, which evaluates the geometry of backbone by analyzing Ramachandran plots [108]. Besides this, the ERRAT server was also tested with non-bonded atom interactions and the possible source of structural aberration realized consequently arriving at a total quality factor of the protein under model [109].

### 4.8. B-Cell Epitope Screening

Ellipro, which is an integrated program in the IEDB-AR v2.22 toolkit, was used to predict the MEV construct conformational B-cell epitopes. Ellipro employs a structure-based approach, where the discontinuous epitopes of the MEV construct are probed on the basis of probing of the three-dimensional structure of the protein. The method is premised on the presence of solvent accessibility and protrusion index values that permit accurate mapping of surface-exposed and immunogenic surfaces that are likely to be recognized by antibodies or B-cell receptors [110]. The analysis provides practical data regarding the structural immunogenicity of the MEV construct and would be a complement to T-cell epitope predictions by showing where areas can cause humoral immune responses to occur.

### 4.9. Molecular Docking Analysis

TLR4 is an essential part of the innate immune system that identifies the antigens of bacteria and activates other downstream signaling to safeguard the host [111]. TLR4 has already been previously reported to be involved in bacterial recognition; therefore, the molecular docking receptor was TLR4 (PDB ID: 2Z62) [72]. The ClusPro 2.0 server has been used in docking simulations that employed clustering and rigid-body docking algorithms which have been able to predict binding configurations that are energetically favorable between the ligand and the receptor [112]. In this regard, default parameters were entered to the PDB structure of MEV construct (ligand) and TLR4 (receptor). Another analysis of the interaction interface was to open the PDBsum server that would display the binding residues and the contact networks of TLR4 receptor and the vaccine construct [113]. The PRODIGY server was additionally utilized in measuring binding affinity and this technique approximates Gibbs free energy change (G 1G 2) of vaccine-receptor complex. In addition, the estimated 0 (Kd) was calculated with the estimated 0 G by means of the thermodynamic equation, G = RT ln (Kd) where R is the universal gas constant and T is the absolute temperature in Kelvins [21]. The data of such computations was extensive on the stability and strength of interaction between MEV and TLR4 with specific attention to the latter being capable of triggering innate immune activation

### 4.10. Normal Mode Analysis of MEV–TLR4 Docked Complex

The intrinsic flexibility and dynamic stability of the MEV-TLR4 complex were determined by using the iMODS server and the Normal Mode Analysis (NMA) with regards to the intrinsic flexibility [114]. NMA was performed with the use of internal coordinates to calculate the overall motions of the docked complex, as well as calculate their conformational motions. iMODS platform can calculate the mobility of macromolecular assemblies and a large range of structural data such as B-factors, eigenvalues, covariance matrices, variance maps, elastic network models, deformability plots, and measures of the amplitude and orientation of motion on a per-residue basis [115]. The MEV construct with TLR4 docked PDB structure was uploaded on iMODS server and defaulted conditions run [22]. This enabled us to predict the compliance of the whole complex, chambers of potential deformation and stability and as a result gain profound knowledge of the molecular dynamics and the flexibility of the interaction between MEV-TLR4.

### 4.11. Molecular Dynamics (MD) Simulations

To further study the structural stability and dynamics of interaction of the docked MEVTLR4 complex, Molecular Dynamics (MD) simulations were done using the GROMACS 2025 package [116]. The best model of docked during the molecular docking was selected to serve as an input to the simulations. To model a closer physiological state, the system was solvated and neutralized by addition of sodium and chloride ions at a concentration of salt of 150 mM. Reduction in energy was realized by the steepest descent algorithm to the convergence of the maximum force (Fmax) to less than 10 kJ/mol, thus producing an energetically stable starting structure. All the covalent bond lengths were fixed to keep the computational efficiency intact in the integration of the algorithm LINCS. Particle Mesh Ewald (PME) method was used to calculate the electrostatics at long range with the cutoff distance of 0.9 nm of the Coulombic and Van der Waals interactions used [117]. The relaxation procedure was performed in two steps in the equilibrated system. The system was initially relaxed through an ensemble simulation of 100 ps to stabilize the system at the constant number of particles (N), the constant volume (V) and the constant temperature (T). This was preceded by a 300 ps NPT ensemble dynamics simulation to equilibrium at constant particle number (N), pressure (P) and temperature (T), which led to density and pressure equilibrium before production dynamics. All simulations were performed using periodic boundary conditions (PBC) of three dimensions (XYZ) to give system continuity. The equilibrated MD production runs were carried out and the obtained trajectories were structurally and energetically analyzed through the GROMACS utilities [118]. Indicators such as root mean square deviation (RMSD), root mean square fluctuation (RMSF), radius of gyration (Rg) and formation of hydrogen bonds were computed to determine stability and compactness of the vaccine receptor complex. The Python 3.11 Matplotlib library was used to graphically represent and plot data, providing the opportunity to interpret the nature and behavior of the interactions and dynamics between the molecules in the course of the simulation in detail [119].

### 4.12. Immune Simulation

The possible immunological profile of the constructed MEV was predicted using the C-ImmSim 10.1 server. The digital tool simulates the dynamics of the mammalian immune system by modeling interactions between key immune compartments such as the bone marrow, lymph nodes, and thymus [21]. For the simulation, the following parameters were used: HLA alleles DRB10101, B0702, and A*0101, a 12,345 random seed value, a volume of 10, a single injection, and 100 steps of the simulation [22]. Such HLA alleles were chosen as they are highly prevalent across the whole world and they are the ones that are essential in antigen presentation such that coverage of population is extensive and an immune response is the best representative of the population that is to be produced. All other things were put on default to offer uniformity and precision of the immune model [120]. Through this type of in silico immune simulation, the capacity of the vaccine to induce primary and secondary immune response such as production of antibodies, release of cytokines and development of memory cells could be assessed. The result was found to be informative on the probable efficacy and immunogenicity of the MEV construct in a model human immune system environment [121].

### 4.13. Reverse Translation, Codon Optimization, and In Silico Cloning

For efficient heterologous expression, the amino acid sequence of the constructed MEV construct was reverse-translated and codon-optimized employing the Java Codon Adaptation Tool (JCat) [122]. A typical host organism for recombinant protein expression, Escherichia coli K-12, was chosen, for which codon optimization was carried out. The analysis provided the key parameters such as Codon Adaptation Index (CAI) and GC content, reflecting translational efficiency and construct stability [75]. A high CAI value (nearly 1.0) and well-balanced GC percentage were believed to be good for higher expression. The vaccine cDNA sequence of the construct was treated with in silico cloning after optimization [82]. Optimized sequence was amplified and cloned into the pET-28a(+) expression vector by SnapGene software 8.0.3 in the right orientation and frame compatibility. Appropriate restriction sites were incorporated for enabling precise cloning, and synonymous codons were changed where required in a manner so as to maintain the natural amino acid sequence [22]. This strategy of codon optimization and cloning enabled MEV construct to be expressed stably in *E. coli* with high efficiency and thus offering a cost-effective system for experimental proof as well as large-scale vaccine production. To address the proteolytic stability of the MEV construct during expression in *E. coli*, intrinsic disorder prediction was evaluated using the IUPred2A server. The analysis showed that the construct is predominantly ordered, with only short flexible regions present at the linker junctions. The EAAAK, GPGPG, and KK linkers incorporated in the design are reported to enhance rigidity, minimize exposure of protease-susceptible sites, and improve solubility, supporting their suitability for *E. coli* expression.

## 5. Conclusions

In this study, a 272-amino-acid MEV construct was rationally designed using peptide linkers and adjuvants to optimize stability and immunogenicity. Physicochemical analyses confirmed its antigenic potential, while structural modeling and validation supported the reliability of the predicted model. Codon optimization further indicated high potential for efficient expression in *E. coli*. Molecular docking and dynamics simulations revealed strong binding with TLR4, and immune simulations predicted robust and long-lasting immune responses. While these findings highlight the promise of the construct against *C. violaceum*, experimental validation remains essential to confirm its efficacy and safety. Overall, this MEV represents a promising step toward novel strategies to address antibiotic resistance and emerging bacterial pathogens.

## Figures and Tables

**Table 1:** Collection of *C. violaceum* strains utilized for the pangenome analysis. The table provides a summary of genomic datasets applied to comparative analysis, with the names of strains, countries of origin, and continents of origin, as well as taxonomic categorization. The strains, taken together, comprise the genetic diversity of *C. violaceum* and were used to build the species-level pangenome for the purpose of subtractive proteomics.

Strain Name	Country	Continent	Organism
*Chromobacterium violaceum* ATCC 12472	–	–	*C. violaceum* ATCC 12472
*Chromobacterium violaceum* CV1192	Brazil	South America	*C. violaceum*
*Chromobacterium violaceum* CV1197	–	–	*C. violaceum*
*Chromobacterium violaceum* CV20	–	–	*C. violaceum*
*Chromobacterium violaceum* FDAARGOS_1273	USA	North America	*C. violaceum*
*Chromobacterium violaceum* FDAARGOS_1274	USA	North America	*C. violaceum*
*Chromobacterium violaceum* FDAARGOS_635	USA	North America	*C. violaceum*
*Chromobacterium violaceum* NCTC9695_assembly	–	–	*C. violaceum*

**Table 2:** Antigenicity, allergenicity, and toxicity analysis of candidate proteins from the *C. violaceum* core proteome. Candidate proteins are shown with accession numbers, functional annotations, and predicted antigenicity scores, as well as allergenicity and toxicity status. These analyses were used for ranking safe and immunogenic proteins for downstream vaccine design.

Accession No.	Protein	Antigenicity	Allergenicity	Toxicity
WP_232514932.1	penicillin-binding protein 1A	0.4664	Non-allergen	Non-toxin
VEB45604.1	Organic solvent tolerance protein	0.6948	Non-allergen	Non-toxin

**Table 3:** Predicted cytotoxic T lymphocyte (CTL) epitopes from some *C. violaceum* proteins. Candidate CTL epitopes from penicillin-binding protein 1A and the organic solvent tolerance protein are displayed with their respective HLA-binding alleles, amino acid positions, antigenicity scores, and immunogenicity values. These epitopes were selected based on antigenic potential, immunogenicity, and good HLA binding for incorporation into the multi-epitope vaccine construct.

Epitope	Protein	Allele	Position	Antigenicity	Immunogenicity
YGYGGTAALPIW	penicillin-binding protein 1A	HLA-C*03:03 HLA-C*12:03 HLA-B*58:01	270–281	1.2146	0.23786
GQYVAEMVRQAM	penicillin-binding protein 1A	HLA-C*14:02	246–357	0.9856	0.01896
GRYGYGGTAALP	penicillin-binding protein 1A	HLA-C*14:02 HLA-C*03:03 HLA-B*48:01	668–679	0.9568	0.19647
GWQGGGGNVTLR	Organic solvent tolerance protein	HLA-B*38:01 HLA-B*48:01	312–323	2.4988	0.15434
LGWQGGGGNVTL	Organic solvent tolerance protein	HLA-B*38:01 HLA-B*48:01	311–322	1.9613	0.13877
PILYSPWLDFPL	Organic solvent tolerance protein	HLA-A*24:02 HLA-E*01:01	165–176	1.8126	0.13888

**Table 4:** *C. violaceum* protein-predicted helper T lymphocyte (HTL) epitopes. Potential HTL epitopes were predicted from penicillin-binding protein 1A and the organic solvent tolerance protein and their HLA-DR binding alleles, amino acid positions, antigenicity scores, and immunogenicity values. Out of the predicted candidates, GRYGYGGTAALPIWI had the greatest antigenicity (1.5906) and immunogenicity (0.4298), revealing its high potential to induce T-helper cell responses and fitness for use in the multi-epitope vaccine construct.

Epitope	Protein	Allele	Position	Antigenicity	Immunogenicity
GRYGYGGTAALPIWI	penicillin-binding protein 1A	HLA-DRB1*07:03	668–682	1.5906	0.4298
LRIDNQGTVPGGEGD	penicillin-binding protein 1A	HLA-DRB1*11:07 HLA-DRB1*03:09 HLA-DRB1*03:05	729–743	1.5661	0.21582
WLNYALGWQGGGGNV	Organic solvent tolerance protein	HLA-DRB1*08:01	306–320	1.3753	0.2801
QGQNQYRVYGSRMTT	Organic solvent tolerance protein	HLA-DRB1*15:02 HLA-DRB1*15:01	114–128	1.0514	−0.27841

**Table 5:** *C. violaceum* predicted B-cell epitopes of proteins. Linear B-cell epitopes were determined from penicillin-binding protein 1A and the solvent tolerance protein. The epitopes are listed with their prediction scores, amino acid positions, antigenicity values, and immunogenicity profiles. The epitope RVKAGDRFRMTRGGDV showed the maximum antigenicity (1.6479) and positive immunogenicity (0.22943), indicating that it has the ability to induce strong humoral immune responses.

Epitope	Protein	Score	Position	Antigenicity	Immunogenicity
DLAGKTGTTSDWKDAW	penicillin-binding protein 1A	0.85	674	1.2111	−0.10202
RVKAGDRFRMTRGGDV	Organic solvent tolerance protein	0.81	87	1.6479	0.22943

## Data Availability

The data presented in this study are available within the article. No additional datasets were generated or analyzed during the current study.

## Supplementary Materials

The following supporting information can be downloaded at: https://www.mdpi.com/article/10.3390/ph19010029/s1, Table S1: Predicted cytotoxic T lymphocyte (CTL), helper T lymphocyte (HTL), and B-cell epitopes derived from *Chromobacterium violaceum* proteins (LptD and Pbp1A) used in constructing the multi-epitope vaccine (MEV) candidate.

## Abbreviations

The following abbreviations are used in this manuscript:

MEV	Multi-Epitope Vaccine
CTL	Cytotoxic T Lymphocyte
HTL	Helper T Lymphocyte
LBL	Linear B Lymphocyte
CTB	Cholera Toxin B Subunit
NCBI	National Center for Biotechnology Information
TLR4	Toll-Like Receptor 4
MHC	Major Histocompatibility Complex
MD	Molecular Dynamics
NMA	Normal Mode Analysis
OMV	Outer Membrane Vesicle
LPS	Lipopolysaccharide
RMSD	Root Mean Square Deviation
RMSF	Root Mean Square Fluctuation
SASA	Solvent Accessible Surface Area
